# Potentially modifiable lifestyle factors, cognitive reserve, and cognitive function in later life: A cross-sectional study

**DOI:** 10.1371/journal.pmed.1002259

**Published:** 2017-03-21

**Authors:** Linda Clare, Yu-Tzu Wu, Julia C. Teale, Catherine MacLeod, Fiona Matthews, Carol Brayne, Bob Woods, CFAS-Wales study team

**Affiliations:** 1 Centre for Research in Ageing and Cognitive Health (REACH), School of Psychology, University of Exeter, Exeter, United Kingdom; 2 PenCLAHRC, Institute of Health Research, University of Exeter Medical School, Exeter, United Kingdom; 3 Centre for Research Excellence in Promoting Cognitive Health, Australian National University, Canberra, Australia; 4 Dementia Services Development Centre Wales, School of Healthcare Sciences, Bangor University, Bangor, United Kingdom; 5 Institute of Health and Society, Faculty of Medicine, Newcastle University, Newcastle, United Kingdom; 6 MRC Biostatistics Unit, Institute of Public Health, University of Cambridge, Cambridge, United Kingdom; 7 Institute of Public Health, University of Cambridge, Cambridge, United Kingdom; University of California San Francisco Memory and Aging Center, UNITED STATES

## Abstract

**Background:**

Potentially modifiable lifestyle factors may influence cognitive health in later life and offer potential to reduce the risk of cognitive decline and dementia. The concept of cognitive reserve has been proposed as a mechanism to explain individual differences in rates of cognitive decline, but its potential role as a mediating pathway has seldom been explored using data from large epidemiological studies. We explored the mediating effect of cognitive reserve on the cross-sectional association between lifestyle factors and cognitive function in later life using data from a population-based cohort of healthy older people.

**Methods and findings:**

We analysed data from 2,315 cognitively healthy participants aged 65 y and over in the Cognitive Function and Ageing Study Wales (CFAS-Wales) cohort collected in 2011–2013. Linear regression modelling was used to investigate the overall associations between five lifestyle factors—cognitive and social activity, physical activity, diet, alcohol consumption, and smoking—and cognition, adjusting for demographic factors and chronic conditions. Mediation analysis tested for indirect effects of the lifestyle factors on cognition via cognitive reserve. After controlling for age, gender, and the presence of chronic conditions, cognitive and social activity, physical activity, healthy diet, and light-to-moderate alcohol consumption were positively associated with cognitive function, together accounting for 20% (95% CI 17%–23%) of variance in cognitive test scores. Cognitive reserve was an important mediator of this association, with indirect effects via cognitive reserve contributing 21% (95% CI 15%–27%) of the overall effect on cognition. The main limitations of the study derive from the cross-sectional nature of the data and the challenges of accurately measuring the latent construct of cognitive reserve.

**Conclusions:**

Cross-sectional associations support the view that enhancing cognitive reserve may benefit cognition, and maintenance of cognitive health may be supported by a healthy and active lifestyle, in later life.

## Abstract 2

In a cross-sectional study, Linda Clare and colleagues investigate whether healthy lifestyle choices are associated with better cognitive function later in life.

## Author summary

**Why was this study done?:**

- Individual differences in lifestyle factors such as physical activity or diet may be related to differences in mental fitness in later life.
- Differences in the extent to which mental fitness declines in later life are thought to arise because some people’s earlier experiences (for example, staying on for further education or playing a leadership role in a job) make their brains more resilient to changes resulting from age or illness—they have higher “cognitive reserve.”
- We wanted to find out whether the concept of cognitive reserve explains how lifestyle influences mental fitness.

**What did the researchers do and find?:**

- We used data from 2,315 mentally fit participants aged over 65 y who took part in the first wave of interviews for the Cognitive Function and Ageing Study Wales (CFAS-Wales).
- Our statistical analyses examined whether a healthy lifestyle (a healthy diet, more physical activity, more social and mentally stimulating activity, moderate alcohol consumption, and refraining from smoking), adjusted to take account of age, gender, and whether people had long-term health conditions, was associated with performance on a test of mental ability.
- When we found an association, we then investigated whether this association was explained by the level of cognitive reserve.
- We found that people with a healthier lifestyle had better mental fitness, and this was partly accounted for by their level of cognitive reserve.

**What do these findings mean?:**

- These results highlight the importance of policies and interventions that encourage older people to make changes in their diet, exercise more, and engage in more socially oriented and mentally stimulating activities.
- Earlier life experiences build cognitive reserve, which helps to maintain mental fitness in later life, so access to education and opportunities to develop skills in the workplace are important in developing this resilience.
- The main limitation of this study is that we used data collected at only one time point, which means that we cannot draw any conclusions about causes or trajectories—we can only say for sure that lifestyle and mental fitness are related.

## Introduction

Cognitive health is a major factor in ensuring the quality of life of older people and preserving independence. Cognitive health is the development and preservation of the multidimensional cognitive structure that allows older people to maintain social connectedness, an ongoing sense of purpose, and the abilities to function independently, to permit functional recovery from illness or injury, and to cope with residual functional deficits [1]. The key components of cognitive health are mental abilities and acquired skills, as well as the ability to apply these so as to engage in purposeful activity [2].

Loss of cognitive health is not an inevitable part of ageing. Some influences on cognitive health, such as gender, genetic profile, history of chronic disease, early life experiences, and the impact of socioeconomic adversity and limited educational opportunity [3,4], cannot be directly modified. Nevertheless, cognitive plasticity—the capacity for enhancement of function in response to altered inputs or environments—is retained to some degree even in later life [5,6]. Furthermore, a systematic appraisal of evidence regarding risk and protective factors for Alzheimer disease has yielded robust evidence for several potentially modifiable lifestyle factors associated with risk level: cognitive activity, social engagement, physical activity, diet, alcohol consumption, and smoking [7]. The contribution of modifiable lifestyle factors to cognitive health means that there may be potential to stabilise or improve declining trajectories of cognitive function. Targeting potentially modifiable lifestyle factors could have positive benefits for cognitive health in later life and serve as a counterweight to elevated genetic risk [8].

In considering the potential for risk reduction, it is important to consider by what mechanisms these lifestyle factors influence cognitive health. Few studies have explored the potential mechanisms involved. Many of the factors identified as relevant to increasing (smoking and high alcohol consumption) or reducing (healthy diet and physical exercise) risk of dementia are equally relevant to other health conditions, particularly through their impact on cardiovascular health [9]. Engagement in cognitive and social activity, however, appears more directly linked to cognitive health.

The concept of cognitive reserve has been proposed to account for individual differences in trajectories of cognitive health and rates of cognitive decline [10]. Cognitive reserve has been defined as the ability of the brain to optimize or maximize performance through differential recruitment of brain networks or use of alternative strategies [10]. Engagement in mental activity—for example, through undertaking education or working in occupations that involve complex demands—is a key determinant of level of cognitive reserve [11,12]. Cognitive reserve reflects the capacity to provide a buffer against the effects of dementia-related brain pathology so that a greater burden of pathology is needed before signs of cognitive decline or symptoms of dementia become evident. It is possible that lifestyle factors may exert their effects on risk by increasing the efficiency of neural networks and hence enhancing cognitive reserve, resulting in greater resilience against the effects of developing neuropathology [13,14]. Cognitive reserve is a latent construct that cannot be directly measured, and assessment therefore relies on proxy indicators. Although cognitive reserve is often indexed by a single proxy measure such as education or IQ, emphasis has recently been placed on the need to combine multiple indicators [15].

This potential pathway via cognitive reserve may help to explain the association between lifestyle factors and cognitive function and thus inform the development of dementia prevention or risk reduction strategies. An appropriate first step is to explore the relationships between these constructs cross-sectionally to determine whether cognitive reserve does indeed play a mediating role. Few empirical studies have investigated this potential mediating pathway, and, in particular, it has seldom been explored in large epidemiological cohorts of older people. Furthermore, most studies have used a single indicator of cognitive reserve, typically education; we could not find any previous studies to date that have used a combined measure of cognitive reserve when examining the relationship between lifestyle factors and cognition [15]. Identifying a mediating role for cognitive reserve in the relationship between current lifestyle factors and cognition is complex because it is likely that past lifestyle will also have influenced these relationships. Therefore, care is needed in selecting appropriate indices to include in a proxy measure of cognitive reserve. In this study, education and occupational complexity were incorporated in a combined proxy measure.

In this cross-sectional analysis, we aimed to explore the potential mediating effect of cognitive reserve, indexed by a combination of educational level and occupational complexity, on the association between lifestyle factors and cognitive function in later life, using data from a large population-based cohort of healthy older people in Wales, United Kingdom. We hypothesized that cognitive reserve would mediate the association between potentially modifiable lifestyle factors (cognitive activity, social engagement, physical activity, diet, alcohol consumption, and smoking) and cognitive function.

## Methods

### Study population

Ethical approval was granted by the North Wales Research Ethics Committee (West), reference number 10/WNo01/37. The Cognitive Function and Ageing Study Wales (CFAS-Wales) is a longitudinal population-based study of people aged 65 y and over in rural (Gwynedd and Ynys Môn) and urban (Neath Port Talbot) areas of Wales that aims to investigate physical and cognitive health in older age and examine the interactions between health, social networks, activity, and participation. Individuals aged 65 y and over were randomly sampled from general medical practice lists between 2011 and 2013, stratified by age to ensure equal numbers in two age groups, 65–74 y and 75+ y. The response rate, in terms of the proportion of those eligible and contactable who participated, was 44%. A further 13% were unable to participate because of ill health. Those who provided written consent to join the study were interviewed in their own homes by trained interviewers and could choose to have the interview conducted through the medium of either English or Welsh. Participants were followed up 2 y later. In this study, we conducted cross-sectional analyses with data from the first wave of interviews (data version 2.0).

While CFAS-Wales is linked to CFAS-II, which was conducted at three sites in England, there are some differences between the two studies in terms of measures used, and, importantly for this study, Wales has over the generations had a somewhat different education system from that of England. The original CFAS included sites in both England and Wales, and the analysis attempted to compensate for these differences, but given that this was already a cross-sectional analysis, it was considered preferable to ensure as homogeneous a population as possible, and hence, we restricted our analyses to CFAS-Wales data.

The baseline sample consisted of 3,593 individuals. For the present analysis, it was important to exclude people with cognitive impairment to avoid potential reverse causality. We excluded anyone with a Mini-Mental State Examination (MMSE) [16] score ≤ 25 (*n* = 908) or an AGECAT [17] classification of dementia (*n* = 185). We also excluded those with an AGECAT classification of depression (*n* = 333), those living in institutions (*n* = 95), those without complete interview data (*n* = 80), and those with missing cognitive test scores (*n* = 4). The sample for this study therefore included 2,315 participants from CFAS-Wales.

### Measures

Cognitive function was measured by the Cambridge Cognitive Examination (CAMCOG), a brief neuropsychological battery designed to assess a range of cognitive functions in the older population, with possible scores ranging from 0–107 [18].

Cognitive reserve was measured by combining two proxy indicators: educational level (years of full-time education) and occupational complexity. Main occupation was recoded using social class and socioeconomic group systems and then reclassified into 15 groups reflecting different levels of occupational complexity [19]. The weights for each component were generated based on the interquartile range to ensure equal contributions to the combined cognitive reserve score, resulting in the following formula:

Cognitive reserve score=1.7×(years of education)+1×(occupational complexity level).

Level of physical activity was determined by the reported frequency of engagement in 18 types of mild (light gardening, bowls, light housework, and home repairs), moderate (gardening, electric lawn mowing, cleaning the car, walking at a moderate pace, dancing, floor or stretching exercises, and heavy housework), and vigorous (jogging, swimming, cycling, aerobics or gym, tennis, heavy gardening, and manual lawn mowing) physical activity. A continuous scale was generated using the frequency levels (0 = once a year or less, 1 = several times a year, 2 = several times a month, 3 = several times a week, and 4 = every day or almost every day) multiplied by the intensity ratio (mild: moderate: vigorous = 1:2:3), which was based on the metabolic equivalent of task (MET) ratio suggested in the literature [20].

Current and exsmokers were identified using two questions: “Do you smoke?” and “Have you ever smoked?”

Self-reported information on the frequency of alcohol consumption over the last 12 mo was used to classify participants into four groups: nearly abstinent (not at all in the last 12 mo or once or twice a year); infrequent drinkers (once or twice a month or once every couple of months); frequent light-to-moderate drinkers (once or twice a week or three or four times a week); and regular light-to-moderate drinkers (five or six times a week or almost every day).

To describe the overall dietary pattern, a total score for healthy diet was generated. CFAS-Wales investigated the frequency of eating (never, seldom, once a week, 2–4 times a week, 5–6 times a week, or daily) and the number of servings per day of fresh fruit, green leafy vegetables, other vegetables, fatty fish, other fish, and wholemeal/brown bread and daily servings of starch foods, dairy foods, and sugary foods. This analysis focused on the frequency of “Mediterranean-style” food intake including fresh fruit, green leafy vegetables, other vegetables, fatty fish, other fish, and wholemeal/brown bread. The frequency included six levels: never, seldom, once a week, 2–4 times a week, 5–6 times a week, or daily. Although evidence has suggested that these are all beneficial components for dementia risk reduction, the amounts and cutoffs selected considerably vary across studies ([21,22]). To describe the overall dietary pattern, a total score for healthy diet was generated based on the six levels of frequency. The range was between 2 (least frequent) and 30 (most frequent), and the mean was 18.2 (standard deviation [SD]: 4.4).

A summary score for cognitive and social activity was generated based on the frequency of seven cognitive (listening to the radio, reading a newspaper, reading a magazine, reading a book, playing games such as cards or chess, doing crosswords, and doing puzzles) and three social activities (“How often do you see any of your [children or other] relatives to speak to?” “Do you attend meetings or any community or social groups?” and “How often do you see any of your neighbours to have a chat or do something with?”). We combined the scores for cognitive and social activity as in many activities cognitive and social elements are closely interlinked.

### Covariates

Information about age, gender, and the presence of chronic conditions was obtained from the interview. Five chronic conditions (hypertension, diabetes, stroke, heart attack, and head injury) were considered to be confounding factors that might influence both lifestyle factors and cognitive function [7,23,24].

### Statistical analysis

The proportion of missing data was small (4%); instances of missing data are documented in Table 1. Comparison of individuals with complete data and those with missing data showed no significant difference in cognitive function. A sensitivity analysis was conducted to investigate the associations in multiple imputation datasets. Distributions were examined prior to finalising the analysis plan. Additional information including results of the sensitivity analysis may be found in the supplemental file (S1 Text).

**Table 1:** Distributions of sociodemographic factors, chronic conditions, and lifestyle factors (*n* = 2,315).

Categorical measures		*n* (%)	Continuous measures	Mean (SD)	Range
Sex	Men	1,132 (48.9)	Age (years)	73.5 0(6.3)	(65–100)
	Women	1,183 (51.1)	Years of education (missing = 6) (years)	12.0 0(2.8)	(1–30)
Chronic conditions (missing = 7)	Hypertension	1,102 (47.7)	Occupational complexity (missing = 63) (level)	8.1 0(3.3)	(1–14)
	Diabetes	384 (16.6)	Cognitive function—CAMCOG (score)	93.4 0(5.4)	(63–105)
	Stroke	124 0(5.4)	Physical activity (missing = 5) (composite score)	19.8 (14.0)	(0–87)
	Heart attack	196 0(8.5)	Diet (missing = 4) (composite score)	18.2 (4.4)	(2–30)
	Head injury	217 0(9.4)	Cognitive and social activity (missing = 12) (composite score)	32.1 (6.2)	(10–49)
Smoking (missing = 9)	Never	981 (42.5)			
	Current smoker	1,128 (48.9)			
	Exsmoker	197 0(8.5)			
Alcohol consumption (missing = 10)	Nearly abstinent	606 (26.3)			
	Infrequent	418 (18.1)			
	Frequent	784 (34.0)			
	Regular	497 (21.6)			

Linear regression modelling was used to investigate the overall associations between each lifestyle factor and cognitive function adjusting for demographic factors and chronic conditions. Since the five lifestyle factors were likely to be correlated, a full model that included all lifestyle factors and covariates was tested.

**Fig 1:**
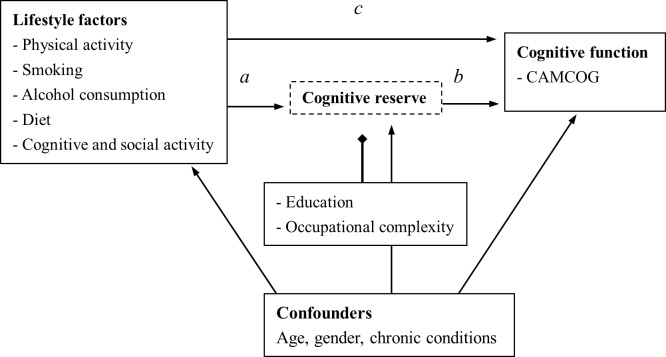
Mediating effect of cognitive reserve on the association between lifestyle factors and cognitive function.

Mediation analysis was used to investigate the mechanisms underlying observed relationships between exposures and outcomes and to examine additional variables hypothesised to be on the causal pathway [22, 23]. Based on the results for the overall associations, the measure of smoking was recategorized into two groups (current versus exsmokers/never) in the mediation analysis. The frequency of alcohol consumption was treated as a continuous variable, and the “trend” (changes in cognitive function per increase in frequency level) was tested in the mediation analysis. To investigate the potential mediating effect of cognitive reserve on the association between lifestyle factors and cognitive function, three pathways (*a*, *b*, and *c*) were estimated using linear regression modelling and adjusting for age, gender, and chronic conditions (Fig 1) [25]. For each lifestyle factor, direct and indirect effects were calculated using the STATA mediation analysis syntax (sgmediation) with bootstrapping confidence intervals [26]. The percentage of indirect pathways among the total effect was calculated to indicate the mediating effect of cognitive reserve on the association between lifestyle factors and cognitive function. All the lifestyle factors were included in one regression model to explore the overall indirect effect of cognitive reserve. Adjusted R-squared was used to indicate the proportion of variance explained by the independent variables. All measures were standardised to provide comparable coefficients across different lifestyle factors.

## Results

Descriptive information for sociodemographic factors, cognitive function, chronic conditions, and lifestyle factors is shown in Table 1. Among the 2,315 participants, the mean age was 74 y (SD: 6.3), and 51% were women. The mean CAMCOG score was 93.4 (SD: 5.4; median: 94; interquartile range (IQR): 7). The average score for cognitive reserve was 28.6 (SD: 6.8), with a range between 9.7 and 62.0.

Table 2 reports the overall association between cognitive function and the potentially modifiable lifestyle factors. Apart from smoking, all the lifestyle factors were significantly associated with cognitive function after adjusting for age, sex, and chronic conditions. Current smoking had negative associations with cognitive function, but the differences did not achieve statistical significance. As shown under model 3 in Table 2, people who reported higher levels of cognitive and social activity (0.20; 95% CI 0.16–0.24), higher levels of physical activity (0.11; 95% CI 0.07–0.15), and healthier dietary patterns (0.13; 95% CI 0.09–0.17) had higher CAMCOG scores. There was a dose-response relationship between cognitive function and frequency of alcohol consumption, with regular light-to-moderate drinkers having higher average CAMCOG scores (0.34; 95% CI 0.23–0.46) than abstainers. In the full model including all the lifestyle factors (Model 4), significant associations with cognitive and social activity, physical activity, healthy diet, and regular light-to-moderate alcohol consumption remained apparent, but the effect sizes slightly reduced. The estimate of adjusted R-squared shows that including all the lifestyle factors explained about 5% of the variation in cognitive function.

**Table 2:** Associations between lifestyle factors and cognitive function.

	Model 1	Model 2	Model 3	Model 4
	Coefficient (95% CI)	Coefficient (95% CI)	Coefficient (95% CI)	Coefficient (95% CI)
Physical activity	0.20 (0.16–0.24)	0.12 (0.08–0.16)	0.11 (0.07–0.15)	0.06 (0.01–0.10)
*p*-value*	<0.01	<0.01	<0.01	0.01
Smoking: Exsmoker versus never	0.11 (0.02–0.20)	0.05 (−0.04 to 0.13)	0.05 (−0.03 to 0.14)	0.02 (−0.06 to 0.10)
Smoking: Current smoker versus never	0.09 (−0.06 to 0.25)	−0.05 (−0.19 to 0.10)	−0.03 (−0.18 to 0.12)	0.05 (−0.09 to 0.20)
*p*-value*	0.04	0.32	0.30	0.77
Alcohol: Infrequent versus nearly abstinent	0.29 (0.16–0.41)	0.19 (0.07–0.31)	0.17 (0.05–0.29)	0.11 (−0.01 to 0.22)
Alcohol: Frequent versus nearly abstinent	0.41 (0.30–0.51)	0.27 (0.16–0.36)	0.24 (0.13–0.34)	0.16 (0.06–0.27)
Alcohol: Regular versus nearly abstinent	0.47 (0.35–0.58)	0.37 (0.26–0.48)	0.34 (0.23–0.46)	0.26 (0.15–0.38)
*p*-value*	<0.01	<0.01	<0.01	<0.01
Diet	0.14 (0.10–0.18)	0.14 (0.10–0.17)	0.13 (0.09–0.17)	0.08 (0.04–0.12)
*p*-value*	<0.01	<0.01	<0.01	<0.01
Cognitive and social activity	0.20 (0.16–0.24)	0.20 (0.16–0.24)	0.20 (0.16–0.24)	0.17 (0.13–0.21)
*p*-value*	<0.01	<0.01	<0.01	<0.01

Model 1: unadjusted; Model 2: adjusted for age and sex; Model 3: adjusted for age, sex, hypertension, diabetes, stroke, heart attack, and head injury; and Model 4: full model including all lifestyle factors, age, sex, hypertension, diabetes, stroke, heart attack, and head injury

*The overall *p*-value for the given lifestyle factor.

Table 3 reports estimates for the three paths a (association between lifestyle factors and cognitive reserve), b (association between cognitive reserve and cognitive function), and c (association between lifestyle factors and cognitive function), as well as the percentage of indirect effect (a to b) among the overall associations. Dietary pattern had the strongest indirect effect (0.05; 95% CI 0.04–0.06) compared to the other lifestyle factors; the indirect effect identified ranged from 36% for diet to 15% for cognitive and social activity. Although smoking showed a potential indirect effect (−0.05; 95% CI −0.09 to −0.02), the association between smoking and cognitive function was not significant.

**Table 3:** Mediation analysis of the effects of cognitive reserve on the association of lifestyle factors with cognitive function.

	Path a (Association between lifestyle factor and cognitive reserve)			Path b (Association between cognitive reserve and cognitive function)			Path c (Association between lifestyle factor and cognitive function)			Indirect effect (a to b)	% of indirect effect
	Model 1	Model 2	Model 3	Model 1	Model 2	Model 3	Model 1	Model 2	Model 3		
	Coefficient	Coefficient	Coefficient	Coefficient	Coefficient	Coefficient	Coefficient	Coefficient	Coefficient	Coefficient	
	(95% CI)	(95% CI)	(95% CI)	(95% CI)	(95% CI)	(95% CI)	(95% CI)	(95% CI)	(95% CI)	(95% CI)	
**Physical activity**	0.11	0.11	0.09	0.25	0.24	0.24	0.17	0.09	0.08	0.02	21%
	(0.07–0.16)	(0.06–0.15)	(0.05–0.14)	(0.21–0.29)	(0.20–0.28)	(0.20–0.28)	(0.13–0.21)	(0.05–0.13)	(0.04–0.12)	(0.01–0.03)	
**Smoking** (current versus never/exsmokers)	−0.20	−0.22	−0.20	0.27	0.25	0.25	0.08	−0.02	−0.01	−0.05	
	(−0.35 to −0.05)	(−0.37 to −0.07)	(−0.35 to −0.06)	(0.23–0.31)	(0.21–0.29)	(0.21–0.28)	(−0.06 to 0.22)	(−0.16 to 0.11)	(−0.15 to 0.12)	(−0.09 to −0.02)	
**Alcohol** (higher versus lower frequency)	0.14	0.14	0.13	0.25	0.23	0.24	0.13	0.09	0.08	0.03	26%
	(0.10–0.17)	(0.10–0.17)	(0.09–0.17)	(0.21–0.29)	(0.20–0.27)	(0.20–0.28)	(0.09–0.16)	(0.05–0.12)	(0.05–0.12)	(0.02–0.04)	
**Diet**	0.21	0.21	0.21	0.25	0.23	0.23	0.09	0.09	0.08	0.05	36%
	(0.17–0.25)	(0.17–0.26)	(0.16–0.25)	(0.21–0.29)	(0.19–0.27)	(0.19–0.27)	(0.05–0.13)	(0.05–0.13)	(0.05–0.12)	(0.04–0.06)	
**Cognitive and social activity**	0.13	0.13	0.13	0.25	0.23	0.23	0.17	0.17	0.17	0.03	15%
	(0.09–0.17)	(0.09–0.18)	(0.09–0.17)	(0.21–0.29)	(0.19–0.27)	(0.19–0.27)	(0.13–0.21)	(0.13–0.21)	(0.13–0.21)	(0.02–0.04)	

Model 1: unadjusted; Model 2: adjusted for age and sex; and Model 3: adjusted for age, sex, hypertension, diabetes, stroke, heart attack, and head injury.

**Fig 2:**
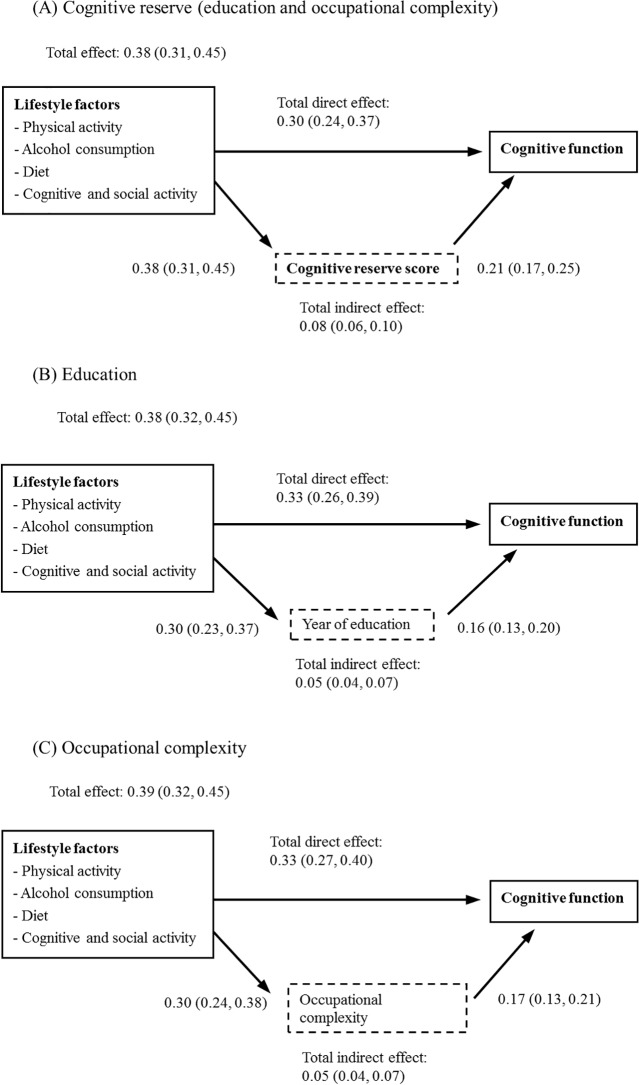
Associations between lifestyle factors, cognitive reserve, and cognitive function (adjusted for age, gender, and chronic conditions).

Four lifestyle factors—cognitive and social activity, physical activity, regular light-to-moderate alcohol consumption, and healthy diet—had both direct (0.30; 95% CI 0.24–0.37) and indirect (0.08; 95% CI 0.06–0.10) associations with cognitive function. The proportion of the total (direct plus indirect) effect of the four lifestyle factors that was mediated by cognitive reserve was 21% (0.08; 95% CI 0.06–0.10) (Fig 2A). This full model explained 20% (adjusted R-square = 0.21) of the variation in cognitive function across the CFAS-Wales participants. Fig 2B and Fig 2C show the mediating effects of individual cognitive reserve components (years of education and occupational complexity) on the associations between cognitive reserve and lifestyle factors. Although the results were similar to those for the combined cognitive reserve score, the effect sizes for the indirect pathways were smaller in these models. The results of sensitivity analysis from the imputed datasets were similar to the main analysis (S1 Text), and therefore, the impact of missing data was small.

## Discussion

This study investigated the potential mediating effect of cognitive reserve on the association between cognitive function and potentially modifiable lifestyle factors through cross-sectional analysis of data from a population-based cohort of older people in Wales. The hypothesis that cognitive reserve plays a mediating role was largely supported. Cognitive and social activity, physical activity, regular light-to-moderate alcohol consumption, and healthy diet were all positively associated with cognitive function and together accounted for 20% of the variance in cognitive test scores. Smoking, however, was not associated with cognitive function. The results of the mediation analysis showed that cognitive reserve, indexed by education and occupational complexity, was an important mediator of the association between the four lifestyle factors and cognition, with indirect effects via cognitive reserve contributing 21% of the overall effect.

This study confirms the relevance of potentially modifiable lifestyle factors for cognition in later life, and, in line with other reports, it emphasizes the possibilities this affords for supporting the maintenance of cognitive health [7,23,27]. Our results are consistent with previous cross-sectional and longitudinal findings on cognitive and social activity. Cognitive activity may reduce risk of dementia [28], while aspects of social engagement are associated with better cognitive function in later life, and possibly with reduced risk of dementia [29]. Similarly, most observational studies of the effects of physical activity on cognition show an association between higher levels of physical activity and lower rates of cognitive decline or dementia [28,30]. Our measure of healthy diet included fruit, vegetable, and fish intake. Research on healthy diets emphasizes the benefits of vegetable consumption and adherence to a Mediterranean-style diet [31–33] as protective of cognitive health, although only oily fish consumption was identified as significant in a systematic review of risk factors [7]. Our findings on alcohol intake are similar to those of studies reporting that light-to-moderate alcohol intake is associated with lower risk than abstaining [34–36], although recent research suggests that while frequent drinking earlier in life is significantly associated with increased risk compared to infrequent drinking, abstaining is not [37]. Smoking, although commonly identified as a risk factor, was not significantly associated with cognitive function in the present study after adjusting for possible confounds.

This study also provides evidence that contributes to explaining the mechanisms underlying the association between these lifestyle factors and cognition and supports the view that cognitive reserve plays an important role in this relationship. Cognitive reserve increases resilience against the effects of neuropathology and hence supports maintenance of function in later life [11]. Cognitive reserve is not a static property but rather is thought to evolve throughout the life course [12], and lifestyle choices may contribute to protecting older people against cognitive decline and dementia by supporting the development, connectivity, and maintenance of brain networks.

The study has several limitations that must be borne in mind. These are cross-sectional data, and hence, we cannot infer causal relationships. Longitudinal follow-up may provide additional information, while comparison of those with high and low cognitive reserve would indicate whether there are differences in lifestyle that distinguish the two groups or alternatively whether cognitive reserve counteracts the effects of a less active cognitive lifestyle. Evaluating these relationships is particularly complex because lifestyle factors such as past engagement in cognitive and social activity may have influenced and contributed to current levels of cognitive reserve. Indeed, some approaches to assessing cognitive reserve include evaluation not only of past but also of current engagement in such activities as part of the proxy cognitive reserve measure [15]. Conceptually, therefore, cognitive lifestyle and cognitive reserve become difficult to distinguish, and this creates challenges for understanding the mechanisms underlying observed associations. We addressed this possible circularity by using only educational level and occupational complexity, two aspects of past experience likely to be relatively stable, in our combined measure of cognitive reserve. As a latent construct, cognitive reserve is difficult to assess accurately, and while evidence suggests that combined proxy measures are more appropriate than single indicators such as educational level, there is as yet no consensus about an optimal approach to measurement. The two indicators we used might be subject to reporting or recall bias or, in the case of occupation, influenced by changing circumstances. Our proxy measure was, therefore, a relatively crude measure. The implication of this is that our findings are likely if anything to underestimate the relationship of cognitive reserve to cognitive function and the extent to which cognitive reserve mediates the association between lifestyle and cognitive function. However, there is a need for greater clarity and consensus about the contributors to and measurement of cognitive reserve and for enhanced study designs that can truly tease out the complexities of the associations between lifestyle factors, cognitive reserve, and cognition.

We excluded people with cognitive impairment to reduce the risk of reverse causality, but it is important to remember that people in the very early stages of cognitive decline may withdraw from social contacts and other types of activity and may change dietary and other habits. Therefore, the potential effects of reverse causality cannot be completely ruled out. Assessment of lifestyle factors was based on self-report during interview and could be subject to bias. In relation to alcohol consumption, the absence of self-reports of heavy drinking or concerns about alcohol in the CFAS-Wales sample in particular might raise questions about possible bias, but it is important to note that only three participants (0.1%) were considered by the interviewer to have a possible drinking problem. Assessment of cognitive function was limited to a global score, and a more fine-grained neuropsychological assessment might reveal more specific associations with particular aspects of cognitive function. There were some missing data, but the extent of this was small and is unlikely to have influenced the findings. Despite these limitations, the particular strength of the study is that it draws on data from a large contemporary population-based cohort of older people in the UK.

### Conclusions

The findings of this study are consistent with the hypothesis that significant associations between four potentially modifiable lifestyle factors—cognitive and social activity, physical activity, healthy diet, and regular light-to-moderate alcohol consumption—and cognition in later life are mediated by level of cognitive reserve. As these findings are derived from cross-sectional data, confirmation from longitudinal analyses will be required. However, these findings provide support for the possibility that enhancing cognitive reserve throughout the lifespan, and encouraging participation in cognitive, social, and physical activity and a healthy diet, may help maintain cognitive health in later life.

## Supporting information

S1 STROBE Checklist Strengthening the Reporting of Observational Studies in Epidemiology (STROBE) Checklist. (DOC)

S1 Text Timeline of analysis plan, rationale for choice of methods, and results of sensitivity analysis using multiple imputation. (DOCX)

## Abbreviations

CAMCOG: Cambridge Cognitive Examination
CFAS-Wales: Cognitive Function and Ageing Study Wales
IQR: interquartile range
SD: standard deviation
STROBE: Strengthening the Reporting of Observational Studies in Epidemiology
