# Supplementary material for: Potentially modifiable lifestyle factors, cognitive reserve, and cognitive function in later life: A cross-sectional study
Source: PLoS Med. 2017 Mar 21;14(3):e1002259. doi: 10.1371/journal.pmed.1002259 (PMC5360216; doi:10.1371/journal.pmed.1002259)
Supplement: S1 STROBE Checklist — (DOC) [file pmed.1002259.s001.doc]

STROBE Statement—Checklist of items that should be included in reports of ***cross-sectional studies***

|  | Item No | Recommendation |
| --- | --- | --- |
| **Title and abstract** | 1 | (*a*) Indicate the study’s design with a commonly used term in the title or the abstract  This study used a cross-sectional design. This is clearly indicated in the title - “Potentially-modifiable Lifestyle Factors, Cognitive Reserve and Cognitive Function in Later Life: a Cross-sectional Study” |
|  |  | (*b*) Provide in the abstract an informative and balanced summary of what was done and what was found  The abstract includes the following sections which clearly detail what was done and what was found in the study: background, method and findings, conclusions. |
| Introduction | | |
| Background/rationale | 2 | Explain the scientific background and rationale for the investigation being reported  This is outlined in the Introduction. For example, in paragraphs 5 and 6:  This potential pathway via cognitive reserve may help to explain the association between lifestyle factors and cognitive function, and thus inform the development of dementia prevention or risk reduction strategies. An appropriate first step is to explore the relationships between these constructs cross-sectionally to determine whether cognitive reserve does indeed play a mediating role. Few empirical studies have investigated this potential mediating pathway and, in particular, it has seldom been explored in large epidemiological cohorts of older people. Furthermore, most studies have used a single indicator of cognitive reserve, typically education; we could not find any previous studies to date that have used a combined measure of cognitive reserve when examining the relationship between lifestyle factors and cognition [15]. Identifying a mediating role for cognitive reserve in the relationship between current lifestyle factors and cognition is complex because it is likely that past lifestyle will also have influenced these relationships. Therefore, care is needed in selecting appropriate indices to include in a proxy measure of cognitive reserve. In this study, education and occupational complexity were incorporated in a combined proxy measure.    In this cross-sectional analysis we aimed to explore the potential mediating effect of cognitive reserve, indexed by a combination of educational level and occupational complexity, on the association between lifestyle factors and cognitive function in later life, using data from a large population-based cohort of healthy older people in Wales, United Kingdom. We hypothesized that cognitive reserve would mediate the association between potentially-modifiable lifestyle factors (cognitive activity, social engagement, physical activity, diet, alcohol consumption and smoking) and cognitive function. |
| Objectives | 3 | State specific objectives, including any prespecified hypotheses  These are stated in the Introduction, paragraph 6, for example:  In this cross-sectional analysis we aimed to explore the potential mediating effect of cognitive reserve, indexed by a combination of educational level and occupational complexity, on the association between lifestyle factors and cognitive function in later life, using data from a large population-based cohort of healthy older people in Wales, United Kingdom. We hypothesized that cognitive reserve would mediate the association between potentially-modifiable lifestyle factors (cognitive activity, social engagement, physical activity, diet, alcohol consumption and smoking) and cognitive function. |
| Methods | | |
| Study design | 4 | Present key elements of study design early in the paper  The design is detailed in the Title, Abstract, Introduction and Method. |
| Setting | 5 | Describe the setting, locations, and relevant dates, including periods of recruitment, exposure, follow-up, and data collection  This information is provided in the Methods section in paragraph 1:  Ethical approval for data collection was granted by the North Wales Research Ethics Committee (West). The Cognitive Function and Ageing Study Wales (CFAS-Wales) is a longitudinal population-based study of people aged 65 and over in rural (Gwynedd and Ynys Môn) and urban (Neath Port Talbot) areas of Wales that aims to investigate physical and cognitive health in older age and examine the interactions between health, social networks, activity and participation. Individuals aged 65 and over were randomly sampled from general medical practice lists between 2011 and 2013, stratified by age to ensure equal numbers in two age groups, 65-74 and 75+. The response rate, in terms of the proportion of those eligible and contactable who participated, was 44%. A further 13% were unable to participate due to ill-health. Those who provided written consent to join the study were interviewed in their own homes by trained interviewers and could choose to have the interview conducted through the medium of either English or Welsh. Participants were followed up two years later. In this study we conducted cross-sectional analyses with data from the first wave of interviews (data version 2.0). |
| Participants | 6 | (*a*) Give the eligibility criteria, and the sources and methods of selection of participants  These are detailed in the Methods section in paragraphs 1 and 2:  Individuals aged 65 and over were randomly sampled from general medical practice lists between 2011 and 2013, stratified by age to ensure equal numbers in two age groups, 65-74 and 75+.  The baseline sample consisted of 3593 individuals. For the present analysis, it was important to exclude people with cognitive impairment to avoid potential reverse causality. We excluded anyone with a Mini-Mental State Examination [MMSE; 16] score <25 (N=908) or an AGECAT (Copeland et al., 1986) classification of dementia (N=185). We also excluded those with an AGECAT classification of depression (N=333), those living in institutions (N=95), those without complete interview data (N=80) and those with missing cognitive test scores (N=4). The sample for this study therefore included 2315 participants from CFAS Wales. |
| Variables | 7 | Clearly define all outcomes, exposures, predictors, potential confounders, and effect modifiers. Give diagnostic criteria, if applicable  This information is detailed in the Methods section, for example in paragraph 10:  Information about age, gender and presence of chronic conditions was obtained from the interview. Five chronic conditions (hypertension, diabetes, stroke, heart attack, and head injury) were considered to be confounding factors which might influence both lifestyle factors and cognitive function [7,20,21]. |
| Data sources/ measurement | 8* | For each variable of interest, give sources of data and details of methods of assessment (measurement). Describe comparability of assessment methods if there is more than one group  This information is provided in the Methods section, paragraphs 3 – 10. For example:  Cognitive function was measured by the CAMCOG, a brief neuropsychological battery designed to assess a range of cognitive functions in the older population, with possible scores ranging from 0 - 107 [17].  Cognitive reserve was measured by combining two proxy indicators: educational level (years of full-time education) and occupational complexity. Main occupation was recoded using social class and socioeconomic group systems and then re-classified into 15 groups reflecting different levels of occupational complexity [18]. The weights for each component were generated based on the interquartile range to ensure equal contributions to the combined cognitive reserve score, resulting in the following formula:  Cognitive reserve score = 1.7 × (education) + 1 × (occupational complexity). |
| Bias | 9 | Describe any efforts to address potential sources of bias  This is discussed in the Methods section, paragraph 11:  The proportion of missing data was small (4%); instances of missing data are documented in Table 1. Comparison of complete cases and those with missing data showed no significant difference in cognitive function. |
| Study size | 10 | Explain how the study size was arrived at  This is explained in the Methods paragraphs 1 and 2. |
| Quantitative variables | 11 | Explain how quantitative variables were handled in the analyses. If applicable, describe which groupings were chosen and why  This is described in the Methods section paragraph 13. For example:  Based on the results for the overall associations, the measure of smoking was re-categorised into two groups (current vs ex-smokers/never) in the mediation analysis. The frequency of alcohol consumption was treated as a continuous variable and the ‘trend’ (changes in cognitive function per increase in frequency level) was tested in the mediation analysis. |
| Statistical methods | 12 | (*a*) Describe all statistical methods, including those used to control for confounding  The statistical methods are described in the Methods section paragraphs 12 and 13. For example:  Linear regression modelling was used to investigate the overall associations between each lifestyle factor and cognitive function adjusting for demographic factors and chronic conditions. Since the five lifestyle factors were likely to be correlated, a full model was tested that included all lifestyle factors and covariates.  Mediation analysis was used to investigate the mechanisms underlying observed relationships between exposures and outcomes and examine additional variables hypothesised to be on the causal pathway [22, 23]. |
|  |  | (*b*) Describe any methods used to examine subgroups and interactions  Not applicable |
|  |  | (*c*) Explain how missing data were addressed  Missing data are discussed in the Methods section paragraph 11.  A sensitivity analysis was conducted to investigate the associations in multiple imputation datasets. |
|  |  | (*d*) If applicable, describe analytical methods taking account of sampling strategy  Not applicable. |
|  |  | (*e*) Describe any sensitivity analyses  These are outlined in the Methods section paragraph 11 (see above). |
| Results | | |
| Participants | 13* | (a) Report numbers of individuals at each stage of study—eg numbers potentially eligible, examined for eligibility, confirmed eligible, included in the study, completing follow-up, and analysed    Sample size is outlined in the Methods section paragraphs 1 and 2 (see above). All results from participants who passed the eligibility criteria were analysed. |
|  |  | (b) Give reasons for non-participation at each stage  Not applicable. |
|  |  | (c) Consider use of a flow diagram  Not applicable. |
| Descriptive data | 14* | (a) Give characteristics of study participants (eg demographic, clinical, social) and information on exposures and potential confounders  Characteristics of participants are presented in Table 1 in the Results section. |
|  |  | (b) Indicate number of participants with missing data for each variable of interest  This is indicated in Table 1 in the Results section. |
| Outcome data | 15* | Report numbers of outcome events or summary measures  Reported in Table 1 in the Results section. |
| Main results | 16 | (*a*) Give unadjusted estimates and, if applicable, confounder-adjusted estimates and their precision (eg, 95% confidence interval). Make clear which confounders were adjusted for and why they were included  This information is provided in Table 3 in the Results section. |
|  |  | (*b*) Report category boundaries when continuous variables were categorized  This information is provided in the Methods section, paragraphs 3 – 10. |
|  |  | (*c*) If relevant, consider translating estimates of relative risk into absolute risk for a meaningful time period  Not applicable. |
| Other analyses | 17 | Report other analyses done—eg analyses of subgroups and interactions, and sensitivity analyses  Reported in paragraph 4 in the Results section, for example:  The results of sensitivity analysis from the imputed datasets were similar to the main analysis and therefore the impact of missing data was small. |
| Discussion | | |
| Key results | 18 | Summarise key results with reference to study objectives  This is provided in the Discussion section, paragraph 1.  This study investigated the potential mediating effect of cognitive reserve on the association between cognitive function and five lifestyle factors through cross-sectional analysis of data from a population-based cohort of older people in Wales. The hypothesis that cognitive reserve plays a mediating role was largely supported. Cognitive and social activity, physical activity, regular light-to-moderate alcohol consumption, and healthy diet were all positively associated with cognitive function, and together accounted for 20% of the variance in cognitive test scores. Smoking, however, was not associated with cognitive function. The results of the mediation analysis showed that cognitive reserve, indexed by education and occupational complexity, was an important mediator of the association between the four lifestyle factors and cognition, with indirect effects via cognitive reserve contributing 21% of the overall effect. |
| Limitations | 19 | Discuss limitations of the study, taking into account sources of potential bias or imprecision. Discuss both direction and magnitude of any potential bias  Limitations are discussed in the Discussion section, paragraphs 4 and 5.  The study has several limitations that must be borne in mind. These are cross-sectional data and hence we cannot infer causal relationships. Longitudinal follow-up may provide additional information, while comparison of those with high and low cognitive reserve would indicate whether there are differences in lifestyle that distinguish the two groups, or alternatively whether cognitive reserve counteracts the effects of a less active cognitive lifestyle. Evaluating these relationships is particularly complex because lifestyle factors such as past engagement in cognitive and social activity may have influenced and contributed to current levels of cognitive reserve. Indeed, some approaches to assessing cognitive reserve include evaluation not only of past but also of current engagement in such activities as part of the proxy cognitive reserve measure [15]. Conceptually, therefore, cognitive lifestyle and cognitive reserve become difficult to distinguish, and this creates challenges for understanding the mechanisms underlying observed associations. We addressed this possible circularity by using only educational level and occupational complexity, two aspects of past experience likely to be relatively stable, in our combined measure of cognitive reserve. As a latent construct, cognitive reserve is difficult to assess accurately, and while evidence suggests that combined proxy measures are more appropriate than single indicators such as educational level, there is as yet no consensus about an optimal approach to measurement. The two indicators we used might be subject to reporting or recall bias or, in the case of occupation, influenced by changing circumstances. Our proxy measure was, therefore, a relatively crude measure. The implication of this is that our findings are likely if anything to underestimate the relationship of cognitive reserve to cognitive function and the extent to which cognitive reserve mediates the association between lifestyle and cognitive function. However, there is a need for greater clarity and consensus about the contributors to and measurement of cognitive reserve, and for enhanced study designs that can truly tease out the complexities of the associations between lifestyle factors, cognitive reserve and cognition.  We excluded people with cognitive impairment to reduce the risk of reverse causality, but it is important to remember that people in the very early stages of cognitive decline may withdraw from social contacts and other types of activity, and may change dietary and other habits. Therefore the potential effects of reverse causality cannot be completely ruled out. Assessment of lifestyle factors was based on self-report during interview and could be subject to bias. In relation to alcohol consumption, the absence of self-reports of heavy drinking or concerns about alcohol in the CFAS Wales sample in particular might raise questions about possible bias, but it is important to note that only 3 participants (0.1%) were considered by the interviewer to have a possible drink problem. Assessment of cognitive function was limited to a global score and a more fine-grained neuropsychological assessment might reveal more specific associations with particular aspects of cognitive function. There were some missing data, but the extent of this was small and is unlikely to have influenced the findings. Despite these limitations, the particular strength of the study is that it draws on data from a large contemporary population-based cohort of older people in England and Wales. |
| Interpretation | 20 | Give a cautious overall interpretation of results considering objectives, limitations, multiplicity of analyses, results from similar studies, and other relevant evidence  Provided in the Discussion section, paragraphs 1, 2, 3, 4 and 5. For example:  The findings of this study are consistent with the hypothesis that significant associations between four potentially-modifiable lifestyle factors – cognitive and social activity, physical activity, healthy diet and regular light-to-moderate alcohol consumption – and cognition in later life are mediated by level of cognitive reserve. As these findings are derived from cross-sectional data, confirmation from longitudinal analyses will be required. However, these findings provide support for the possibility that enhancing cognitive reserve throughout the lifespan, and encouraging participation in cognitive, social and physical activity and a healthy diet, may help maintain cognitive health in later life. |
| Generalisability | 21 | Discuss the generalisability (external validity) of the study results  This is mentioned in the Discussion section, paragraph 5.  Despite these limitations, the particular strength of the study is that it draws on data from a large contemporary population-based cohort of older people in the United Kingdom. |
| Other information | | |
| Funding | 22 | Give the source of funding and the role of the funders for the present study and, if applicable, for the original study on which the present article is based  Detailed in the additional submission required information. |

*Give information separately for exposed and unexposed groups.

**Note:** An Explanation and Elaboration article discusses each checklist item and gives methodological background and published examples of transparent reporting. The STROBE checklist is best used in conjunction with this article (freely available on the Web sites of PLoS Medicine at http://www.plosmedicine.org/, Annals of Internal Medicine at http://www.annals.org/, and Epidemiology at http://www.epidem.com/). Information on the STROBE Initiative is available at www.strobe-statement.org.
