# Supplementary material for: Potentially modifiable lifestyle factors, cognitive reserve, and cognitive function in later life: A cross-sectional study
Source: PLoS Med. 2017 Mar 21;14(3):e1002259. doi: 10.1371/journal.pmed.1002259 (PMC5360216; doi:10.1371/journal.pmed.1002259)
Supplement: S1 Text — (DOCX) [file pmed.1002259.s002.docx]

**ANALYSIS TIMELINE**

| **Date** | **Action** |
| --- | --- |
| December 2015 | Initial analysis plan outlined |
| 18 December 2015 | Data requested |
| 17 February 2016 | Preliminary dataset received (version 1) |
| March 2016 | Data examined for distribution of variables, and analysis plan updated |
| April 2016 | Cognitive reserve score calculated |
| 17 May 2016 | Proposed analysis run with preliminary dataset |
| 8 August 2016 | Final dataset received (version 2.0) |
| 10 August 2016 | Analysis run with final dataset |
| September 2016 | Analysis reported for inclusion in paper |
| 18 November 2016 | Paper submitted |
| December 2016 | Sensitivity analyses run as requested by reviewer to address issue of missing data (response 2 to reviewer 3 in the rebuttal) |
| 19 December 2016 | Revised version of paper submitted |

**RATIONALE FOR CHOICE OF METHODS**

**Hypotheses**

In this cross-sectional analysis we aimed to explore the potential mediating effect of cognitive reserve, indexed by a combination of educational level and occupational complexity, on the association between lifestyle factors and cognitive function in later life, using data from a large population-based cohort of healthy older people in Wales, United Kingdom. We hypothesized that cognitive reserve would mediate the association between potentially-modifiable lifestyle factors (cognitive activity, social engagement, physical activity, diet, alcohol consumption and smoking) and cognitive function.

**Analysis plan (December 2015)**

**Analysis steps:** The hypothesis will be answered by use of a structural equation model. The initial steps develop the relationship of the component parts prior to fitting them together in the mediation model. The initial steps investigate a) the extent to which the lifestyle factors of physical activity, diet, smoking, and alcohol intake predict the cognitive lifestyle score which indexes cognitive reserve (comprising education, occupation, cognitive activity and social contact); (b) the extent to which the lifestyle factors of cognitive activity, social contact, physical activity, diet, smoking and alcohol intake predict cognitive function; and (c) the extent to which the cognitive lifestyle score predicts cognitive function. Linear regression analysis will be undertaken to investigate these relationships. Lifestyle factors that are seen to be related (p<0.1) in part a and b will be included in the final model. Cognitive reserve will also be checked to ensure it is related to cognitive function. Combining the lifestyle factors and cognitive reserve using a linear structural equation model with both direct (from lifestyle to cognition) and indirect (from lifestyle to cognition, through cognitive reserve) factors will be considered influential with significance level p=0.05. Potential confounders of age, gender and the presence of any of 5 chronic co-morbid conditions - hypertension, diabetes, stroke, heart attack, and head injury – which may affect cognitive function will be adjusted for without statistical selection in a step-wise manner to investigate their relationship on the model coefficients.

**Sample to be used:** To ensure that we minimise the impact of reverse causality individuals will be excluded who demonstrate cognitive impairment, or for whom there is a need for continuous care. Individuals will be excluded if they have a MMSE score <25 or an AGECAT classification of dementia or depression, or are living in residential care. We will also exclude participants who do not have complete measurements on our risk factors or for whom cognitive test scores are missing. A comparison of individuals excluded for missing data versus the complete cases will be undertaken to evaluate potential missing data bias.

**Variables:** Cognitive reserve will be assessed using a cognitive lifestyle score; this will comprise measures of education and occupational complexity (question 40, years of education; questions 45 – 50, occupation).

Assessment of cognitive function will be based on responses to the CAMCOG (questions 266 – 372).

The lifestyle factors (other than cognitive and social activity) to be considered will be physical activity, diet, smoking and alcohol intake. Physical activity is covered in questions 375 – 395, which give a measure of number of activities undertaken and frequency of activity for the categories of vigorous, moderate and light activity. Diet is covered in questions 191 – 200, covering intake of oily fish, fruit and vegetables. Smoking is covered in questions 500 – 505 which indicate current and past smoking behaviour; other substance use is covered in questions 506 – 507. Alcohol intake is covered in questions 508 – 529.

**Calculation of the cognitive lifestyle score:** The cognitive lifestyle score relates to two component parts of an individual’s life. These are education history (investigated using number of years of full time education), and occupational complexity (measured using social class, and social economic group of main employment). A combination score has previously been used extensively (e.g. Valenzuela et al. 2011 – reference 18 in paper) and we have adopted a similar approach for this analysis. The years of education variable has a range of 8-32 years, but the majority of the population lie between 9 years and 14 years as indicated from leaving school at statutory level through to postgraduate qualifications. Social class and occupational complexity are combined using 14 categories, indicated by highly professional, high social class occupations as 1 (such as lawyer with employees, hospital consultant) through to 14 (such as cleaner, advertising sign holder). Category 15 is used for divorced housewives with no history of occupation. To ensure that the spread of both parts of the score are of equal value the score will be calculated by a weighted sum of the two components with the weights determined by the interquartile ranges (IQR) of the two components, One component will be weighted 1, the other component will be weighted as the ratio of the two IQRs. The distribution of the final variable will be inspected to ensure a continuous scale with large range of values. During the analysis stage this item will be standardised.

**Actual analysis (August 2016)**

Initial steps, data checking and review of methods proposed

During this stage we investigated the missing data, by variable, and compared complete cases and those with missing data. We reviewed the distributions of variables. The cognitive lifestyle score was calculated.

Linear regression modelling

Linear regression modelling was used to investigate the overall associations between each lifestyle factor and cognitive function adjusting for demographic factors and chronic conditions. Since the five lifestyle factors were likely to be correlated, a full model was tested that included all lifestyle factors and covariates to ensure sufficient independent effects.

Adjustment of variables based on preliminary modelling

The measure of smoking was re-categorised into two groups (current vs ex-smokers/never) in the mediation analysis, to reduce the number of parameters.

The frequency of alcohol consumption was treated as a continuous variable and the ‘trend’ (changes in cognitive function per increase in frequency level) was identified as appropriate for use in subsequent analyses and reduced the number of parameters.

Mediation analysis

To investigate the potential mediating effect of cognitive reserve on the association between lifestyle factors and cognitive function, three pathways (*a, b, c*) were estimated using linear regression modelling and adjusting for age, gender and chronic conditions. For each lifestyle factor, direct and indirect effects were calculated using the STATA mediation analysis syntax (sgmediation) with bootstrapping confidence intervals. The percentage of indirect pathways among the total effect was calculated to indicate the mediating effect of cognitive reserve on the association between lifestyle factors and cognitive function. All the lifestyle factors were included in one regression model to explore the overall indirect effect of cognitive reserve. Adjusted R-squared was used to indicate the proportion of variance explained by the independent variables. All measures were standardised (within the programme) to provide comparable coefficients across different lifestyle factors.

**Sensitivity analysis** **(December 2016)**

In response to reviewer request, we conducted a sensitivity analysis using multiple imputation replicating the mediation analysis. The imputation model included all factors included in the final model for all individuals in our sample (i.e. excluding the cognitively frail). The regression coefficients from imputed datasets were similar (within 2 decimal places) to the results of complete case analysis. We can therefore conclude that the 4% missing data had limited impact on the findings. Results of the sensitivity analysis are shown in the Appendix.

**RESULTS OF SENSITIVITY ANALYSIS**

Results of complete case analysis and multiple imputation (MI)

|  | Path a (Association between lifestyle factor and cognitive reserve) | | Path b (Association between cognitive reserve and cognitive function) | | Path c (Association between lifestyle factor and cognitive function) | |
| --- | --- | --- | --- | --- | --- | --- |
|  | Main analysis | MI results | Main analysis | MI results | Main analysis | MI results |
| **Cognitive reserve** | - | - | 0.21  (0.17, 0.25) | 0.20  (0.16, 0.24) | - | - |
| **Physical activity** | 0.02  (-0.02, 0.07) | 0.03  (-0.02, 0.07) | - | - | 0.05  (0.00, 0.09) | 0.05  (0.01, 0.09) |
| **Alcohol** (higher vs lower frequency) | 0.10  (0.06, 0.13) | 0.10  (0.06, 0.13) | - | - | 0.07  (0.03, 0.10) | 0.07  (0.03, 0.10) |
| **Diet** | 0.17  (0.13, 0.22) | 0.17  (0.13, 0.21) | - | - | 0.04  (0.00, 0.08) | 0.04  (0.00, 0.08) |
| **Cognitive and social activity** | 0.09  (0.04, 0.13) | 0.09  (0.04, 0.13) | - | - | 0.15  (0.11,0.19) | 0.15  (0.11, 0.19) |
